# Bilateral Primary Intraocular Lymphoma

**Published:** 2011-10

**Authors:** Mehrdad Karimi, Masoud Soheilian, Mozhgan Rezaei Kanavi

**Affiliations:** 1 Ophthalmic Research Center, Shahid Beheshti University of Medical Sciences, Tehran, Iran; 2 Central Eye Bank of Iran, Tehran, Iran

**Keywords:** B-cell Lymphoma, Primary Intraocular Lymphoma

## Abstract

**Purpose:**

To report a case of bilateral primary intraocular lymphoma.

**Case report:**

A 33-year-old man presented with bilateral blurred vision since two years ago. Examination revealed large keratic precipitates, anterior chamber reaction, posterior subcapsular cataracts, and vitreous infiltration. After a short trial of topical and periocular steroids, diagnostic 25-gauge pars plana vitrectomy was performed and cytologic evaluation of the aspirate confirmed a diagnosis of intraocular lymphoma. The patient was subsequently managed with intravitreal methotrexate in both eyes and responded favorably. Central nervous system workup for lymphoma was negative.

**Conclusion:**

Primary intraocular lymphoma should be considered in young adults suffering from chronic recalcitrant panuveitis.

## INTRODUCTION

Malignant lymphomas are categorized into Hodgkin and non-Hodgkin lymphomas (NHL). Although most NHLs (80%) arise from B-lymphocytes and their precursor cells, non-Hodgkin lymphomas originating from T-lymphocytes (14%) and natural killer cells (6%) have also been well-recognized. Primary central nervous system lymphoma (PCNSL) may arise from the brain, spinal cord, meninges or eyes. PCNSL constitutes 1-2% of all lymphomas and 5% of all primary CNS tumors.^1^ Primary intraocular lymphoma (PIOL) is an uncommon subset of PCNSL which involves the retina, vitreous or optic nerve head.^2^,^3^ PIOL typically affects elderly patients at a mean age of 60 years and rarely occurs in young children.^1^ Herein, we report a young patient with bilateral PIOL masquerading as panuveitis for a couple of years.

## CASE REPORT

A 33-year-old Caucasian male with no significant past medical history, was referred to our center in June 2010 with refractory bilateral panuveitis of 2 years’ duration. He was being treated with fluorometholone 0.1% drops every 6 hours, tropicamide 1% every 8 hours and prednisolone 50 mg per day. Best corrected visual acuity (BCVA) was counting fingers at 30cm (20/3200) in both eyes. Slit lamp examination revealed multiple large keratic precipitates (KPs) and deep anterior chamber with +1 cell reaction in both eyes. There was mild posterior subcapsular (PSC) cataract in the right eye but the crystalline lens was clear in the left eye. There were 2+ cells in the anterior vitreous in both eyes. Intraocular pressure (IOP) was 15 mmHg and 13 mmHg in the right and left eyes, respectively. Due to hazy media, details were not visible but the retina seemed attached. Systemic and laboratory evaluations for uveitis including complete blood count (CBC), erythrocyte sedimentation rate (ESR), C-reactive protein (CRP), tuberculin skin test (PPD), angiotensin converting enzyme (ACE), urine analysis, chest X-ray, HLA-B5, HLA-B51 and HLA-B27 which had been done at another center were negative. Preliminary diagnosis was panuveitis suspecting a masquerade syndrome.

**Figure 1:**
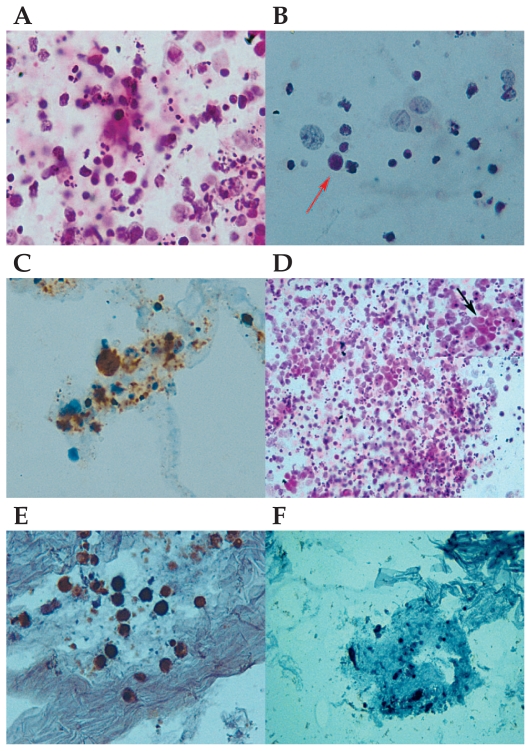
Cytopathologic features of the right vitreous sample: **(A)** Note the heterogenous lymphoid infiltration, some with medium to large hyperchromatic nuclei (Hematoxylin & Eosin, ×1000). **(B)** A moderately large-sized mononuclear cell (arrow) with hyperchromatic and irregular nucleus (Hematoxylin & Eosin, ×1000) and **(C)** a few large cells with strong immune reactivity for CD20 (×1000). Cytopathologic findings in the left vitreous sample: **(D)** note the heterogenous infiltration of lymphoid cells (Hematoxylin & Eosin, ×400) composed of clusters of medium to large-sized cells (arrow in small box on the upper right side) with hyperchromatic nuclei. **(e)** Note strong immune reactivity of the atypical cells for CD20 (×1000). **(F)** Lack of immune reactivity for CD3 (×1000).

Trans-septal triamcinolone acetonide was injected in the right eye and five days later diagnostic 25-gauge pars plana vitrectomy and intravitreal triamcinolone acetate (2 mg) injection was performed in his left eye. Ten days later pars plana vitrectomy and intravitreal injection of methotrexate (400microgram/0.1ml) was performed in the right eye; at the same session, intravitreal methotrexate was also injected in the left eye. Diluted and undiluted vitreous samples were processed for cytopathology. Cytospin and cytoblock preparations of specimens from the right eye disclosed a heterogenous infiltration of cells, some with medium to large hyperchromatic nuclei and scattered ghost cells (Figures 1A and 1B). Most of the cells were lost during immunocytochemistry but still a few large cells which were immunoreactive for CD20 were present (Figure 1C). These cytopathologic features in the right eye were suggestive of intraocular B-cell lymphoma. Cytopathologic examination of specimens from the left vitreous revealed a heterogenous infiltration of lymphoid cells among which clusters of medium to large cells with hyperchromatic nuclei were observed (Figure 1D). The cells were strongly immunoreactive for CD20 (Figure 1E) but not for CD3 (Figure 1F). CD3 is a cell marker specific for T-lymphocytes and since PIOL originates mainly from B-cells, a positive immune reaction for CD3 is not seen in PIOL. The cytopathologic findings in left eye were also suggestive of intraocular B-cell lymphoma and supported the previous cytopathologic diagnosis in the fellow eye.

Neuroimaging studies and neurological consultation was performed. Brain and spinal cord MRI was negative for lymphoma. During 15 months of follow up, the patient received two injections of intravitreal methotrexate (400microgram/0.1ml) in his right eye, 3 and 5 months and one in his left eye, 4 months after surgery. At final examination, BCVA was improved to 4/10 and 3/10 in the right and left eyes respectively and intraocular inflammation was well controlled.

## DISCUSSION

Many patients with the intraocular variant of PCNSL are initially misdiagnosed as having uveitis. The patient described herein had PIOL masquerading as panuveitis for 2 years. In a series of 32 histologically proven cases of PIOL, the average interval between the onset of ocular symptoms and histological diagnosis was 21 months.^1^ Although PIOL usually affects elderly patients, it should be considered in any patient with chronic uveitis. As an example in 1994, two young patients with intraocular lymphoma presented with retinal vasculitis.^4^ Intraocular lymphoma generally occurs at a younger age in HIV-positive subjects.^5^ As a result of the varied clinical features and rarity of the condition, a high index of suspicious is required for the diagnosis of PIOL. Early diagnosis is important since it enables rapid initiation of treatment, may reduce morbidity and improve the prognosis.

It is difficult to arrive at a histopathological diagnosis of PIOL,^6^–^8^ thus research has been focused on developing other methods to assist the diagnosis of this condition. These include immunohistochemistry, flow cytometry, molecular analysis, cytokine level analysis, and polymerase chain reaction based methods.^9^–^11^ The actual incidence PIOL is unknown. The incidence of PCNSL has increased in both immunocompetent and immunocompromised subjects from 0.027/100,000 in 1973 to 1/100,000 in the early 90s.^12^ The underlying cause for the increase in immunocompetent patients is unknown.^13^

Characteristically, PIOL causes bilateral involvement in up to 80% of cases. Ocular involvement may precede, occur simultaneously or follow CNS disease. More than 50% of PCNSL cases present with ophthalmic findings. The most common manifestations of PIOL are isolated posterior uveitis or vitritis (50% of cases), combined anterior and posterior uveitis (22% of cases) and choroiditis, chorioretinitis or subretinal pigment epithelial infiltrates.^1^ A histological diagnosis should be established by performing cytologic examination of vitreous samples obtained by pars plana vitrectomy.

Most patients with ocular NHL die within 2 years of diagnosis as a result of progressive or recurrent CNS disease. Previous reports suggested that approximately 80% of patients with PIOL will subsequently develop brain lymphoma.^6^,^7^

AIDS-related immunosuppression and congenital or iatrogenic immunodeficiencies are risk factors for NHL. However, no clear risk factors have been identified for PCNSL in immunocompetent individuals. In our patient the presence of refractory uveitis for two years, negative laboratory tests and a high index of suspicious prompted diagnostic vitrectomy. If vitreous biopsy is unable to establish the diagnosis, biopsy of retinal and choroidal lesions can be performed; this is a potentially hazardous intervention which can be achieved by a variety of external and internal approaches.^14^ This, however, should be considered as the investigation of last resort. Biopsy samples should be taken only after consultation with histologists, microbiologists and molecular biologists to obtain advice on tissue handling, culture and fixation methods.

Ever since the first description of intraocular (non-Hodgkin) lymphoma as ocular reticulum cell sarcoma in 1951,^15^ its treatment has evolved from enucleation^16^ to radiation therapy alone or in combination with chemotherapy, systemic and intravitreal chemotherapy, and more recently to biological therapy. The optimal goal of treatment for vitreoretinal lymphoma is eradication of intraocular disease on one hand and prevention of CNS or systemic relapse, on the other. The most efficacious therapy has not yet been identified and reports are limited to small case series. The drug of choice for intravitreal chemotherapy may be methotrexate (400 μg). In order to overcome the side effects and reduce the frequency of intravitreal injections of methotrexate and also to effectively deal with intraocular lymphomas resistant to methotrexate therapy, intravitreal injections of rituximab (1mg/0.1ml) for treatment of vitreoretinal lymphoma has been introduced.

Rituximab is a mouse/human chimeric immunoglobulin G1-K monoclonal antibody that targets the CD20 antigen present on the surface of malignant and normal B-lymphocytes, and received FDA approval in 1997 for systemic use in patients with follicular non-Hodgkin lymphoma and diffuse large B-cell lymphoma. It has also been used for other types of B-cell lymphoma. Since the vast majority of PIOLs and PCNSLs are B-cell in origin and express the CD20 antigen,^17^,^18^ rituximab is an ideal drug for intraocular and intrathecal adjuvant therapy.

In recent years, researchers have investigated innovative methods for treatment of intraocular lymphoma in animal models. Gregory and associates^19^ studied a novel treatment for intraocular lymphoma using membrane Fas ligand vesicles, the membrane-only form of Fas ligand, to activate innate immunity and terminate the immune privilege of the eye.

In summary this case report reveals that PIOL may occur in young adults and making an early diagnosis may save the patient’s sight.
